# A Neutralizing Monoclonal Antibody Targeting the Acid-Sensitive Region in Chikungunya Virus E2 Protects from Disease

**DOI:** 10.1371/journal.pntd.0002423

**Published:** 2013-09-12

**Authors:** Suganya Selvarajah, Nicole R. Sexton, Kristen M. Kahle, Rachel H. Fong, Kimberly-Anne Mattia, Joy Gardner, Kai Lu, Nathan M. Liss, Beatriz Salvador, David F. Tucker, Trevor Barnes, Manu Mabila, Xiangdong Zhou, Giada Rossini, Joseph B. Rucker, David Avram Sanders, Andreas Suhrbier, Vittorio Sambri, Alain Michault, Marcus O. Muench, Benjamin J. Doranz, Graham Simmons

**Affiliations:** 1 Blood Systems Research Institute, San Francisco, California, United States of America; 2 Department of Laboratory Medicine, University of California, San Francisco, San Francisco, California, United States of America; 3 Integral Molecular, Philadelphia, Pennsylvania, United States of America; 4 Queensland Institute of Medical Research, Brisbane, Queensland, Australia; 5 DIMES, Microbiology, University of Bologna, Bologna, Italy; 6 Purdue University, West Lafayette, Indiana, United States of America; 7 Centre Hospitalier Universitaire, Groupe Hospitalier Sud-Réunion, La Reunion, France; Centers for Disease Control and Prevention, United States of America

## Abstract

The mosquito-borne alphavirus, chikungunya virus (CHIKV), has recently reemerged, producing the largest epidemic ever recorded for this virus, with up to 6.5 million cases of acute and chronic rheumatic disease. There are currently no licensed vaccines for CHIKV and current anti-inflammatory drug treatment is often inadequate. Here we describe the isolation and characterization of two human monoclonal antibodies, C9 and E8, from CHIKV infected and recovered individuals. C9 was determined to be a potent virus neutralizing antibody and a biosensor antibody binding study demonstrated it recognized residues on intact CHIKV VLPs. Shotgun mutagenesis alanine scanning of 98 percent of the residues in the E1 and E2 glycoproteins of CHIKV envelope showed that the epitope bound by C9 included amino-acid 162 in the acid-sensitive region (ASR) of the CHIKV E2 glycoprotein. The ASR is critical for the rearrangement of CHIKV E2 during fusion and viral entry into host cells, and we predict that C9 prevents these events from occurring. When used prophylactically in a CHIKV mouse model, C9 completely protected against CHIKV viremia and arthritis. We also observed that when administered therapeutically at 8 or 18 hours post-CHIKV challenge, C9 gave 100% protection in a pathogenic mouse model. Given that targeting this novel neutralizing epitope in E2 can potently protect both in vitro and in vivo, it is likely to be an important region both for future antibody and vaccine-based interventions against CHIKV.

## Author Summary

CHIKV is characterized by acute and chronic polyarthritis/polyarthralgia that can be debilitating and protracted. Currently there are no FDA-approved vaccines or specific antiviral treatments for CHIKV. We thus identified and characterized human monoclonal antibodies directed against CHIKV that could be utilized in prophylactic and immediate post-exposure settings. Such patient derived monoclonal antibodies could also provide useful information on critical antigens and epitopes for development of future vaccines and other biologics. We describe here the identification of two monoclonal antibodies (C9 and E8) isolated from recovered patients. C9 potently inhibited CHIKV infection in cells and prevented viremia and arthritis in a mouse model of CHIKV disease. The epitope for this antibody includes an amino-acid residue in a key acid-sensitive region of the E2 glycoprotein of CHIKV. Rearrangement of this region following exposure to low pH is critical for uncovering portions of the companion E1 glycoprotein, required for successful entry of CHIKV into cells. We hypothesize that binding of antibodies to this region stabilizes the native complex and thus prevents such rearrangements.

## Introduction

Chikungunya virus (CHIKV) is a mosquito-borne alphavirus first isolated in Tanzania in 1952 [1] that has caused sporadic outbreaks of predominantly rheumatic disease every 2–50 years, primarily in Africa and Asia. The largest epidemic of CHIKV disease ever recorded took place during 2004–2011, and involved an estimated 1.4 to 6.5 million cases and the first autochthonous CHIKV infections in Europe (Italy in 2007 and France in 2010) [2], [3]. Imported cases were also reported in nearly 40 countries, including European countries, Japan, and the USA. The epidemic was associated with the emergence of a new clade of viruses, which were efficiently transmitted by *Aedes albopictus*, a mosquito vector that has seen a dramatic global expansion in its geographic distribution [4]–[6]. CHIKV disease is characterized by acute and chronic polyarthritis/polyarthralgia, which is usually symmetric and often incapacitating and occasionally protracted [4]–[8]. Other symptoms, such as fever, rash, myalgia, and/or fatigue, are often also present during the acute phase. The recent epidemic was also associated with atypical and severe clinical forms of CHIKV disease and some fatalities, which appeared to be restricted to the very young and elderly patients with comorbidities [8], [9].

CHIKV virions contain three major structural proteins: glycosylated El and E2 envelope (env) proteins embedded in the viral membrane, and a non-glycosylated nucleocapsid protein. Based on similarity to other alphaviruses, E2 mediates receptor attachment, while E1 is a class II viral fusion protein. A third glycoprotein, E3, is associated with mature virions in some alphaviruses [10], but not others [11], while 6K protein, a membrane-associated peptide created by cleavage of the polyprotein to release E2 and E1, is incorporated into particles at a low level [12], [13].

The organization of alphavirus surface glycoproteins in virions has been defined using cryo-electron microscopy (cryo-EMs) [14], while the atomic structure of CHIKV glycoproteins was recently solved by x-ray crystallography [15], both for mature particles and for immature p62 Env precursor polyprotein prior to furin cleavage. 240 copies each of three glycoproteins (E3/E2/E1) come together to form a protein coat with icosahedral symmetry and containing 80 spikes [15]. The folding, transport to the surface and function of these glycoproteins relies on their correct interactions with each other. E1 consists of three β-sheet domains, termed I, II and III, while E2 contains three immunoglobulin-like domains (A, B and C, with A being at the N-terminus). In the complex, domain B lies at the membrane distal end and contacts E3, domain C is closest to the viral membrane and domain A is in the center. E1 interacts laterally with E2 all along domain II, along with additional points of contact from other regions of E1. E1 contains an internal fusion loop at the tip of domain II, which in the mature structure exists as a β-hairpin lodged in a groove between domains A and B of E2 [15]. E3 also plays a role in protecting the fusion loop from premature exposure.

Treatment of CHIKV rheumatic disease usually involves non-steroidal anti-inflammatory drugs (NSAIDs) and/or simple analgesics, which can provide relief but is often inadequate [8]. Although a number of vaccine strategies have been, or are being, explored [16]–[19], there are currently no licensed human vaccines [20]. Nevertheless, it is clear that CHIKV neutralizing antibodies from infected humans or vaccinated monkeys can mediate protection prophylactically, or soon after exposure. Polyclonal immunoglobulins derived from humans recovered from CHIKV infection, when passively transferred into neonatal and interferon α/β receptor deficient (IFNAR^−/−^) mice, protected these animals from CHIKV-induced viremia and mortality [21]. Purified total IgG from monkeys immunized three times with a CHIKV virus-like-particle vaccine (containing E1 and E2) similarly protected IFNAR^−/−^ mice from CHIKV viremia and mortality [17]. A recent study described two monoclonal antibodies (mAbs), 5F10 and 8B10, which were isolated from CHIKV infected individuals. These mAbs specifically neutralized CHIKV and o'nyong'nyong virus (ONNV, a virus closely related to CHIKV), but none of the other alphaviruses tested [22]. The 5F10 and 8B10 mAbs, when used in escape mutant studies were shown to recognize key residues in E2 (V216) and E1 (T101), respectively [23]. The combination of 5F10 and 8B10 were also shown to significantly delay CHIKV-driven lethality in mice deficient in IFNα/β and IFNγ receptors, and mature B and T cells [24]. Similarly, a group of mouse-derived mAbs, clustering close to the putative receptor-binding domain of E2 [25], were also found to be protective to various degrees in mouse models of CHIKV [26]. Additionally, an immunodominant linear epitope at the N-terminus of E2 is also a target for protective antibodies [27].

Herein, we describe the isolation and characterization of two human mAbs, C9 and E8, from patients that were infected with CHIKV and recovered. We also report the characterization of antibody binding epitopes using a library of alanine scanning mutants of CHIKV envelope covering 910 residues (>98%) of the CHIKV E1 and E2 glycoproteins. Although the binding epitopes for C9 and E8 both mapped to CHIKV E2, C9 was able to potently neutralize CHIKV, while E8 was not. The neutralizing epitope bound by C9 mapped to the acid-sensitive region (ASR) that is critical for the rearrangement of CHIKV E2 during fusion and viral entry into host cells. Purified human C9 antibody, when used prophylactically in an adult wild-type mouse model of CHIKV disease, completely protected against viremia and arthritis.

## Materials and Methods

### Ethics statement

Written informed consent was obtained from recovered CHIK donors in Italy and Reunion, France and collection complied with relevant human subjects research protocols approved by the institutional review boards of the University of Bologna and the Centre Hospitalier Universitaire, respectively. Animal work was conducted in accordance with good animal practice (NHMRC, Australia), and was approved by the QIMR animal ethics committee. Additional murine studies were performed at Blood Systems Research Institute with approval of the Institutional Animal Care and Use Committee at ISIS Services LLC (San Carlos, CA) and following the recommendations of the National Research Council's Institute of Laboratory Animal Resources as published in their Guide for the Care and Use of Laboratory Animals.

### Generation of C9 and E8 IgG mammalian expression constructs

CHIKV mAb C9 variable chains were sequenced by MC Labs (South San Francisco, CA). For mammalian expression, C9 variable heavy (VH) and light (VL) chain cDNAs were synthesized by Genscript (Piscataway, NJ). The closest human germline signal sequences (ss), VH5 5a and VKIII A27, were used to ensure efficient processing and secretion. SS-VH cassettes were cloned into a pCAGGS mammalian expression vector as EcoRI-NheI fragments, upstream of the human IgG1 heavy chain constant region. SS-VL cassettes were cloned as EcoRI-BsiWI fragments upstream of the human kappa light chain constant region. CHIKV FAb CAP101A.E8 variable heavy and light chain cDNAs bearing human IL-2 signal sequences were synthesized by Genscript. IL-2ss-VH and IL-2ss-VL cassettes were cloned as MfeI-NheI and MfeI-BsiWI fragments upstream of their respective constant regions, as described above.

### CHIKV wild-type envelope pseudovirion production

CHIKV envelope (E3/E2/E1) in a pCAGGS vector was used for pseudoparticle preparation as described previously [28]. Lentiviral pseudotypes were produced essentially as described [29] by using 10 µg of luciferase reporter plasmid, (pNL-luc, based on pNL4-3-R-E-) [30] and 30 µg of plasmid encoding viral envelope. Virions were concentrated by ultracentrifuge concentration at 100,000×g in a SW28 rotor (Beckman) through a 20% sucrose cushion for 1.5 h at 4°C. The pellets were resuspended overnight in HBSS at 4°C.VSV-G and alphavirus envelopes expressing the RRV, SFV and SINV were used as controls for pseudovirion neutralization assay [31].

### CHIKV wild-type pseudovirus neutralization assay

HEK 293T cells were plated at 2×10^4^ cells/well in DMEM (HyClone) containing additives and incubated at 37 C in 5% CO_2_ overnight. The following day, serial dilutions of antibody and virus pre-incubated for 45 min were added to the HEK 293T cells. A spin infection was performed at 1,200×g for 60 min and cells incubated for an additional 3 hours at 37°C. The antibody-virus mix was removed by aspiration and replaced with 100 µl of pre-warmed fresh media. The cells were incubated for 48 hrs before samples were recovered for measurement of luciferase activity in the cell lysates as per manufacturers protocol (Promega).

### CHIK wild-type virus production, plaque assay and 80% PRNT assay

CHIKV was obtained from ATCC (ATCC # vr-64), from a strain originally isolated in 1953 from the serum of a patient in East Africa and expanded in suckling mice. Replication competent CHIKV was grown in Vero cells. Vero cells (0.5×10^5^) were plated in a 6-well (Costar) plate overnight. Serial dilution of the virus stock (250 µl) was incubated with cells for 1 hr at 37°C. One hour after incubation, an overlay of 4% agarose (Life Technologies) in DMEM supplemented with 2% FBS was added to cells and incubated at 37°C for 72 hrs. Subsequently, wells were fixed with 4% formaldehyde and stained with 0.1% crystal violet in methanol: ethanol. Plaques were counted against a white background.

Vero cells (0.5×10^5^) were plated in a 6-well (Costar) plate overnight. Serially diluted monoclonal antibodies were mixed with CHIK live virus diluted to 400 PFU/ml and pre-incubated for an hour at 37°C. Following this 250 µl of the antibody-virus mixture was added to the confluent Vero cell monolayer for an additional hour. Subsequently, the virus was removed and an overlay of 4% agarose in DMEM supplemented with 2% FBS was added and cells were incubated at 37°C for 72 hrs. The plaques were stained and counted as described above. The PRNT titer is calculated as the reciprocal of the serum dilution, where ≥80% reduction in the number of plaques is compared to the negative control in the presence of media and no mAbs.

### Isolation of anti-CHIK-V antibody from EBV transformed B cells

The PBMCs for EBV transformed B cell isolation were obtained from two CHIKV infected and recovered individuals. B cells were isolated using the Miltenyi MACS Switched Memory B cell Isolation kit (130-093-617) according to the manufacturer's protocol. The cells were plated at 30 cells per well in 96 U-bottom plates. PBMC from unrelated donors were treated with Mitomycin C and used as feeder cells at 5×10^4^ cells per well. The cells were cultured in RPMI supplemented with 7% FBS, 1000 IU/L IL-2 (Roche) and 2.5 µg/ml R848 peptide (InvivoGen) [32]. Filtered B95-8 EBV supernatants (diluted 1 in 3) were added to each well and incubated for one week before being replaced with fresh media. EBV transformed B cell supernatants expressing CHIKV specific antibodies were screened for CHIKV pseudotype neutralization potential [28]. The cells from the positive wells were clonally isolated by limiting dilution followed by expansion and cloning.

### Immune phage antibody library construction

An immune FAb phage display library was constructed from peripheral blood donated by three CHIKV-infected individuals. All three individuals were infected in Réunion Island, France, during the 2006 outbreak. Peripheral blood samples were drawn 2–3 years after infection and serum was analyzed for the presence of neutralizing antibodies using CHIKV reporter virus pseudotypes. Total RNA was prepared using Tri-Reagent (Sigma) with standard protocols. RNA was converted to cDNA using Super Script First-Strand Synthesis System for RT-PCR (Invitrogen) following the manufacturer's instructions. Construction of the library was performed by GenScript (Piscataway, NJ) as previously described [33]. The final library was transformed into *E.coli* TG1 cells (Invitrogen) using electroporation, and the quality of the library was assessed by sequence analysis of 100 randomly picked clones.

### Characterization of antibody binding kinetics using biosensor

All biosensor studies were performed at 25°C using a ForteBio Octet Red biosensor system (ForteBio, Menlo Park, CA). CHIKV VLPs were loaded onto amine-reactive biosensor tips (AR2G) using an immobilized human antibody against CHIKV (E26D9.02, a gift from Dendritics, Lyon, France). Briefly, AR2G tips were activated for 5 minutes with a mixture of 20 mM EDC (1-ethyl-3-(3-dimethylaminopropyl)carbodiimide hydrochloride, Sigma, St. Louis, MO) and 10 mM sulfo-NHS (N-hydroxysulfosuccinimide, Sigma, St. Louis, MO) in water. E26D9.02 diluted to 25 µg/ml in 10 mM sodium acetate, pH 5.5, was allowed to react for 10 minutes and then the tips were deactivated for 5 minutes with 1 M ethanolamine (Sigma, St. Louis, MO). After a brief rinse, CHIKV VLPs diluted to 20 µg/ml were loaded for 45 minutes followed by a 10 minute stabilization. Tips were then transferred to PBS buffer supplemented with 1 mg/ml BSA (PBS-B) for subsequent antibody binding studies. C9 was prepared as a two-fold serial dilution (starting at 20 µg/ml) plus buffer blanks. Antibody association was measured for 5 minutes followed by a 50 minute dissociation in buffer. Non-specific binding was assessed using sensor tips without VLPs. Data analysis was performed using Octet Data Analysis v6.4 (ForteBio, Menlo Park, CA). Binding kinetics were analyzed using a standard 1∶1 binding model.

### Shotgun mutagenesis mapping studies

A CHIKV Env expression construct (S27 strain) with a C-terminal V5 tag was subjected to high-throughput alanine scanning mutagenesis to generate a comprehensive mutation library. Primers were designed to mutate each residue within the E2, 6K, and E1 regions of Env to alanine, while alanine codons were mutated to serine. In total, 910 CHIKV Env mutants were generated (98.5% coverage), sequence confirmed, and arrayed into 384-well plates. Each Env mutant was transfected into HEK-293T cells and allowed to express for 22 hrs. Cells were stained for 1 h with human mAbs C9 (0.42 µg/ml), E8 (2 µg/ml), CKV061 (0.75 µg/ml, isolated from phage display library in identical manner to E8), E26D9.02 (0.5 µg/ml, a gift from Dendritics), and rabbit polyclonal antibody (1∶2000 dilution, a gift from IBT Bioservices) diluted in 10% NGS (Sigma). mAbs were detected using 3.75 µg/ml AlexaFluor488-conjugated secondary antibody in 10% NGS (Jackson ImmunoResearch Laboratories) for 1 h. Mean cellular fluorescence was detected using the Intellicyt high throughput flow cytometer (HTFC, Intellicyt). Antibody reactivities against each mutant Env clone were calculated relative to wild-type env protein reactivity by subtracting the signal from mock-transfected controls and normalizing to the signal from wild-type Env-transfected controls. Mutations were identified as critical to the epitope if they did not support reactivity of the test human mAb, but did support reactivity of the other CHIKV antibodies. This counter-screen strategy facilitates the exclusion of env mutants that are misfolded or have an expression defect [34]. Critical amino acids required for antibody binding were visualized on CHIKV env crystal structures (monomer PDB ID #3N41 and trimer PDB ID #2XFC, [15]), to obtain 3D epitope maps.

### CHIKV mouse model; arthritis and viremia monitoring

Female C57BL/6 mice (6 weeks old) were inoculated with CHIKV as described previously [35]. Briefly, mice were inoculated with CHIKV {10^4^ log_10_ 50% cell culture infectivity dose (CCID_50_)} in 40 µl RPMI 1640 (supplemented with 2% fetal calf serum) by shallow subcutaneous injection into the top, towards the lateral side, of each hind foot in the metatarsal region, injecting toward the ankle. Mice (n = 4 mice per group) were injected with (i) PBS; (ii) purified C9 mAb or (iii) purified control human mAb at 0.5 mg/mouse by the intraperitoneal route one day (day −1) prior to infection on day 0 with CHIKV. In order to avoid stimulating non-specific immune responses that may interfere with CHIKV infection of adult mice [35], C9 and control antibodies with endotoxin levels below 10 EU/mg were used. Arthritis was monitored by measuring the height and width of the metatarsal area of the hind feet using digital calipers [35]. The data is presented as a group average of the percentage increase in foot height×width for each foot compared with the same foot on day 0. Viremias were measured by collecting 40 µl of blood from a tail vein into 0.8-ml MiniCollect serum separation tubes (Greiner Bio-One GmbH, Kremsmunster, Austria). The tubes were spun at 4,000×g for 2.5 min on a bench-top microcentrifuge. Serum was collected and viral titers were determined as described previously and expressed as CCID_50_ per ml [35].

### CHIKV neonate mouse experiment

The ability of antibodies to protect against the lethal effects of CHIKV infection were evaluated in a murine model as previously described [21]. All animal experiments were performed with approval of the Institutional Animal Care and Use Committee at ISIS Services LLC (San Carlos, CA). Briefly, C57BL/6J mice were purchased from Jackson Laboratories (Sacramento, CA) and bred at BSRI. Breeder pairs were housed under specific-pathogen free conditions in micro-isolator cages (Innovive Inc., San Diego, CA). Mice were checked daily and the date when litters were first observed was considered day 0. On day 9, litters with their mothers were transferred to static disposable cages (Innovive Inc.) and transferred a BSL-3 facility for infection and treatment. Neonatal C57BL/6J mice were infected with 5×10^5^ PFU of CHIKV (S27 strain) intradermally in the ventral thorax. Some mice were also intraperitoneally injected with C9 or control human IgG/mouse in 0.2 ml phosphate-buffered saline (PBS) immediately prior to CHIKV infection. The control IgG used in this experiment was purified IgG from human serum (Sigma-Aldrich). Results were analyzed using Kaplan-Meier survival curves using Aable version 3.06 software (Gigawiz Ltd., Co., OK). The significance of differences was determined using log-rank chi-square analysis with the results not adjusted for multiple comparisons. Results are considered significant if *P*≤0.05.

## Results

### Isolation of human neutralizing antibodies

The anti-CHIKV human monoclonal antibody C9 was isolated by EBV transformation of B cells from a CHIKV infected and recovered individual identified during a 2007 outbreak of CHIKV in Northern Italy [36]. CHIKV pseudovirus [28] neutralization was used as the primary screening assay for the selection of B cell clones and heavy and light chains were subsequently sequenced from the clones. Separately, a Fab fragment (E8) was isolated from a phage display library constructed from multiple CHIKV infected and recovered individuals from the 2005–6 epidemic on La Réunion as described in the materials and methods. A virus-like particle (VLP) binding assay, using VLPs produced from CHIKV capsid and E3/E2/E1 envelope (env) glycoprotein expression was used as the primary screen for panning phage, followed by use of the CHIKV pseudovirus (HIV- backbone based, without CHIKV capsid) neutralization assay for downstream characterization. Subsequently, the antibody heavy and light chains for C9 and E8 were sequenced and cloned into human full length IgG vectors for protein production and evaluation.

### Potent *in vitro* neutralization

**Figure 1:**
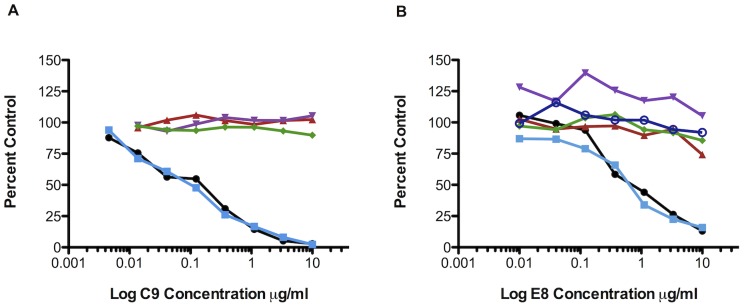
Human mAbs C9 and E8 neutralize CHIKV pseudovirions. Neutralization of pseudovirus bearing CHIKV S27 wild-type (black); CHIKV A226V mutant (light blue); SFV (red); SINV (magenta); RRV (green) and VSV (dark blue) envelope by (**A**) C9 or (**B**) E8 mAbs. Antibody concentration is shown in the x-axis. The results are expressed as the percentage of no antibody control and represent mean of triplicate wells, and is representative of three experiments.

C9 and E8 were tested in neutralization assays performed in HEK 293T cells using CHIKV pseudoviruses bearing an envelope from the prototypical West African, Asian, and East/Central/South African (ECSA) CHIKV strain, S27. The C9 and E8 IgG antibodies neutralized CHIKV pseudoviruses at approximately 0.1 µg/ml and 1.0 µg/ml (IC_50_) respectively (Figure 1). Pseudoparticles produced using envelopes derived from the LR2006 OPY-1 strain from the La Reunion outbreak were similarly sensitive to neutralization, with IC_50_ values of 0.4 µg/ml and 10 µg/ml for C9 and E8 respectively (Figure S1). Similar neutralization was observed regardless of the cell type used (data not shown). Neutralization was specific to CHIKV, with no detectable cross-reactivity to pseudoviruses expressing other alphavirus envelopes from RRV, SFV and SINV, as well as VSV-G (Figure 1). The mAb also neutralized CHIKV envelopes with a naturally occurring mutation at a critical site near the fusion loop in E1 (A226V) that is associated with increased CHIKV infectivity for, and transmission by, the mosquito vector, *Aedes albopictus* (C9, IC_50_ 0.1 µg/ml; E8, IC_50_ 1.0 µg/ml for S27[A226V]) (Figure 1) [37].

When tested in a replication competent CHIKV plaque reduction neutralization test (PRNT) using the S27 strain, C9 exhibited a PRNT80 value of approximately 0.3 µg/ml. A comparable level of neutralization was also observed with the LR2006 OPY-1 strain. In contrast to the weak neutralization observed with the CHIKV pseudovirus assay (Figure 1), E8 failed to neutralize replication competent CHIKV, even at concentrations up to 20 µg/ml. Similarly, little to no inhibition by E8 was noted utilizing vesicular stomatitis virus-based pseudotypes (rather than HIV-based) or in a cell-cell fusion assay, while C9 maintained similar neutralizing and inhibitory activity (data not shown). Based on these findings, C9 can be categorized as a strongly neutralizing antibody, with similar potency to other human mAbs [22], while E8 is a non-neutralizing, or weakly neutralizing, antibody of live virus.

### Binding properties of anti-CHIKV human mAbs

**Figure 2:**
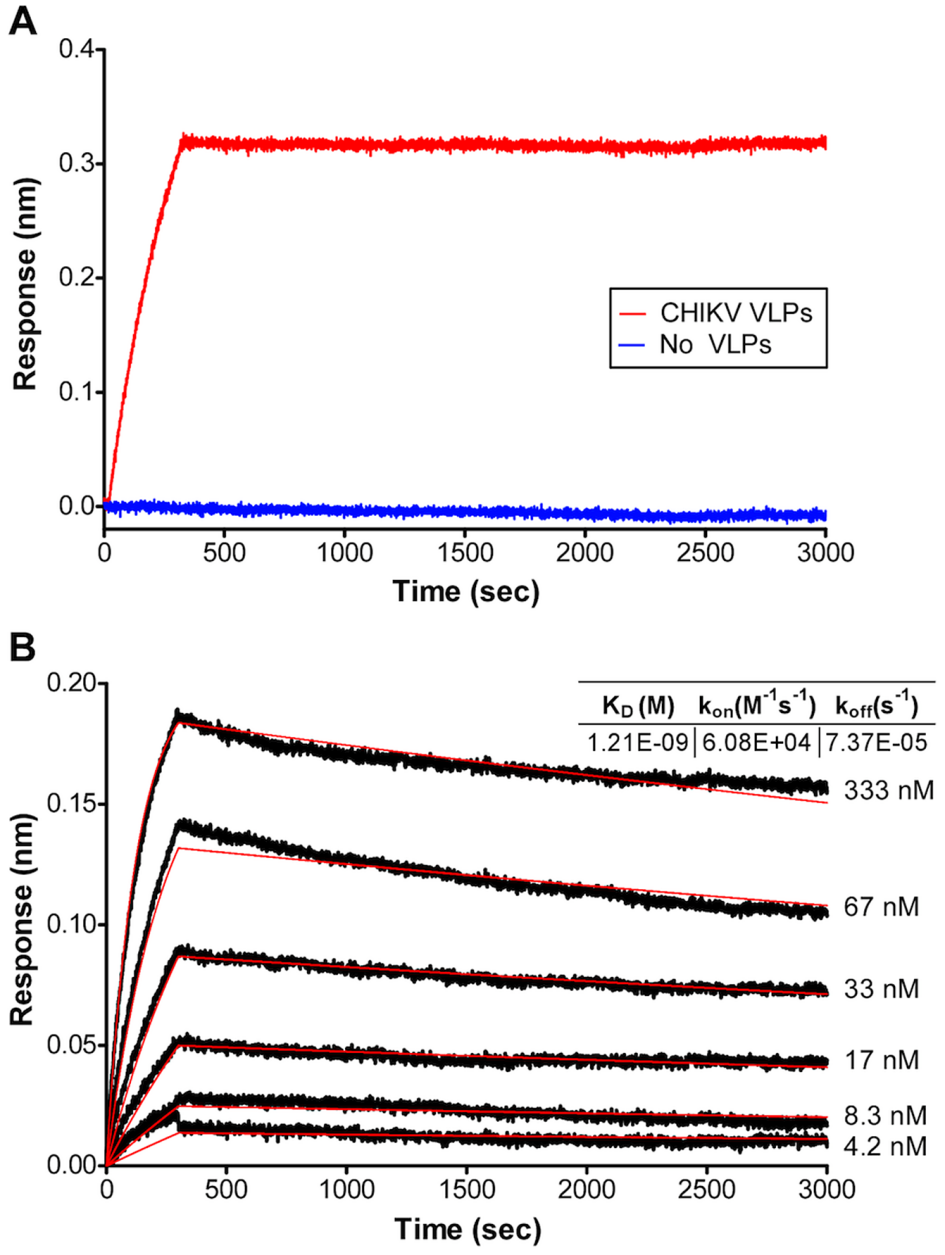
Kinetic analysis of binding to intact CHIKV virus like particles. (**A**) Summary of antibody/antigen interactions. Binding of anti-CHIKV antibody C9 to intact CHIKV VLPs was detected using the FoteBio OctetRed biosensor. C9 mAb (66.6 nM) binding to CHIKV VLPs or a non-particle surface control is used to show binding specificity of mAb to intact CHIKV VLP. (**B**) C9 dose response curve for binding intact CHIKV VLPs. Raw data curves for antibody associating and dissociating from captured CHIKV VLPs are shown in black and fitted curves are shown in red. Data were fitted to a 1∶1 binding model to determine association rate (k_on_) and dissociation rate (k_off_), and equilibrium binding affinity (K_D_) was calculated. C9 binds CHIKV VLPs with 1.21 nM apparent affinity.

In order to determine how strongly each mAb interacts with the native virion, intact CHIKV VLPs were captured onto the surface of ForteBio Octet RED biosensor tips (ForteBio, Menlo Park, CA) and antibody binding to the immobilized particles was measured using BioLayer Interferometry (ForteBio, Menlo Park, CA). Whereas C9 bound to VLPs with an apparent affinity of 1.2 nM (Figure 2A & B), E8 failed to recognize CHIKV envelope protein on intact VLPs (data not shown), suggesting that the E8 epitope may be occluded in the native E1/E2 conformation on virions. This finding is consistent with the inability of E8 to neutralize live CHIKV. C9 and E8 antibodies recognized envelope derived from CHIKV VLPs under semi-native conditions (protein run in SDS-PAGE gels without reducing agent), suggesting that both C9 and E8 recognize conformation specific epitopes that are dependent on disulfide bonds (Figure S2).

### mAb epitope mapping using Shotgun Mutagenesis

In order to identify residues in the binding epitope of C9 and E8, the mAbs were screened against a comprehensive CHIKV mutation library in which nearly every residue within the E2, 6K, and E1 envelope subunits (encompassing 910 amino-acid residues with 98.5% coverage) were individually mutated to an alanine (alanines were mutated to serines). Each clone was expressed in HEK-293T cells and assessed for C9 and E8 antibody binding using immunofluorescence staining. Mean fluorescence was determined by high-throughput flow cytometry and antibody reactivity to each mutant was calculated relative to reactivity to wild-type (WT) CHIKV env. Clones were identified as critical for binding if they had low reactivity to C9 or E8 but high reactivity to other CHIKV E2-specific control antibodies (CKV061, E26D9.02, and rabbit polyclonal antibody, described in materials and methods). This counter-screen strategy facilitates the exclusion of env mutants that are globally or locally misfolded or that have an expression defect [34]. Residues identified in this way are the energetically critical contributors of an epitope, the so-called ‘hot-spots’ of mAb binding [38], [39].

**Figure 3:**
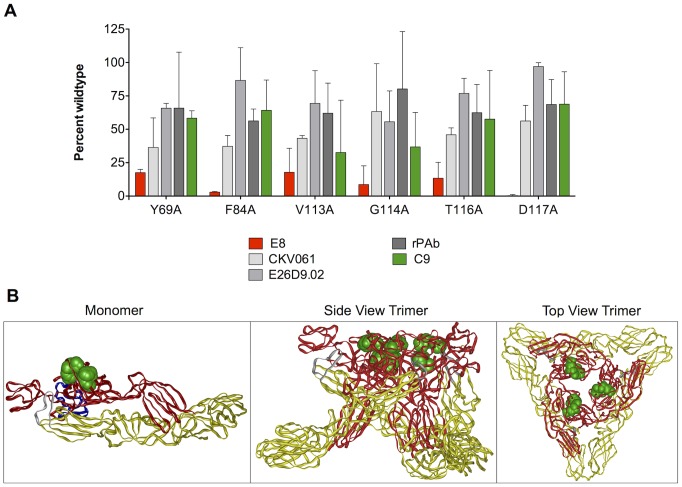
Critical residues and predicted E8 binding site. (**A**) HEK-293 cells expressing mutant CHIKV envelope proteins were immunostained with E8 antibody. Clones with reactivity <20% relative to wild-type CHIKV env were identified as critical for E8 binding. Mutation of six individual E2 residues to alanine (Y69, F84, V113, G114, T116, and D117) significantly reduced E8 binding (red bars) but did not affect binding of C9 (green bar) or other control antibodies (gray bars). Residues are numbered according to E2 in PDB entry #3N41 [15]. (**B**) Critical binding residues for E8 (shown in green) were visualized on the CHIKV env crystal structure. The E1, E2, and E3 envelope protein subunits in the monomer (PDB Entry #3N41) are depicted in yellow, red, and blue, respectively and the fusion loop is shown in silver (left panel). In the side-view and top-down trimeric representations (center, and right panels, PDB entry #2XFC), E3 is not in the structure. In the side view trimeric representation (center panel), the viral membrane is positioned at the bottom of the figure.

Six amino acids clustered within the E2 Domain A were identified as critical for E8 binding. Residues E2-Y69, E2-F84, E2-V113, E2-G114, E2-T116, and E2-D117, when mutated to alanine, all reacted at less than 20% of WT reactivity when screened with E8, but had high reactivity compared to three other antibodies (CKV061, E26D9.02 and rabbit polyclonal antibody), suggesting that the mutant envelope proteins are expressed and properly folded (Figure 3A). The E8 epitope appears to be partially occluded when visualized on the native trimer structure (Figure 3B), which likely accounts for the poor neutralization exhibited by E8.

### C9 antibody binding residue mapped to the acid-sensitive region of E2

**Figure 4:**
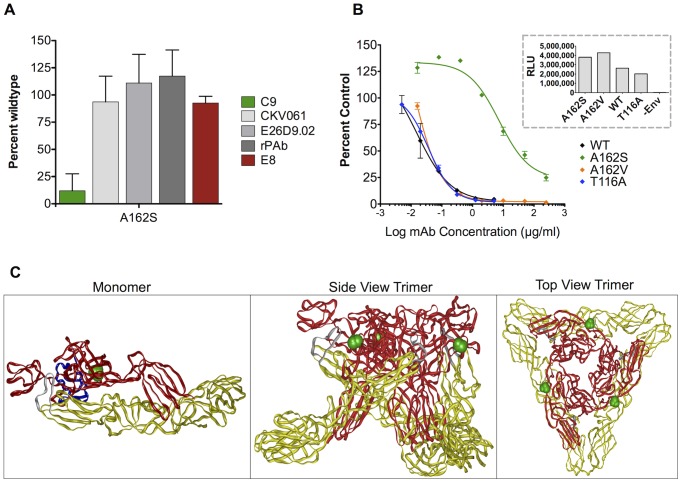
Critical residues and predicted C9 binding site. (**A)** HEK-293 cells expressing mutant CHIKV envelope proteins were immunostained with C9 antibody. Clones with reactivity <20% relative to wild-type CHIKV env were identified as critical for C9 binding. Mutation of residue A162 in E2 to serine significantly reduced C9 binding (green bar) but did not affect binding of E8 (red bar) or other control antibodies (gray bars). Residues are numbered according to E2 in PDB entry #3N41 [15]. (**B**) A162S and A162V pseudoviruses were tested for C9 inhibitory potency. The infectivity of the mutants compared to WT was tested (inset graph), indicating that the mutants did not hinder CHIKV env folding or function. Average raw luminescence units are shown for each construct and an env-minus negative control. (**C**) The critical residue A162 (shown in green) was visualized on the CHIKV env crystal structure. The E1, E2, and E3 env protein subunits in the monomer (PDB Entry #3N41) are depicted in yellow, red, and blue, respectively and the fusion loop is shown in silver (left panel). In the side-view and top-down trimeric representations (center and right panels, PDB entry #2XFC), E3 is not in the structure. In the side view trimeric representation (center panel), the viral membrane is positioned at the bottom of the figure.

Similar epitope mapping studies using Shotgun Mutagenesis alanine scanning identified residue E2-A162, located in the β-connector region between domains A and B of CHIKV E2, as a critical residue required for C9 recognition (Figure 4). The E2-A162 residue is solvent exposed and is predicted to be easily accessible when CHIKV Env is in the native trimer conformation (Figure 4). The E2-A162 residue is in the acid-sensitive region (ASR), sandwiched in a critical pocket between CHIKV E1, E2 and E3, as determined by the CHIKV envelope crystal structure [15], [40]. Interestingly, the ASR, along with the E2 domain B, was also recently described for alphaviruses as being unstructured following acid pH triggering [40]. In our study we found that residue E2-A162, when mutated to serine, reacted at 12% of WT reactivity against C9 but reacted at greater than 70% of WT reactivity against other anti-CHIKV antibodies, strongly suggesting that the E2-A162S mutant is properly folded and involved in the C9/envelope binding interaction (Figure 4A). Other residues are also likely to be involved in the C9 epitope, but either contribute weakly to the interaction or their individual mutation to alanine does not sufficiently disrupt mAb binding to be detected as ‘critical’.

Using pseudovirions, no virus entry defects were observed with E2-A162S, further indicating that the mutant envelope is properly folded. To confirm the importance of this residue in C9 binding, infection experiments were conducted with wild type and mutant pseudovirions. E2-A162S pseudovirions were inefficiently neutralized by C9, with a 490-fold increase in the C9 IC_50_, demonstrating that this residue is required for potent C9 inhibition (Figure 4). In contrast, wild type E2 and E2-A162V, a naturally occurring variant [41], remained fully sensitive to C9.

### C9 mAb inhibited viremia and arthritis in an adult wild-type mouse model of CHIKV disease

**Figure 5:**
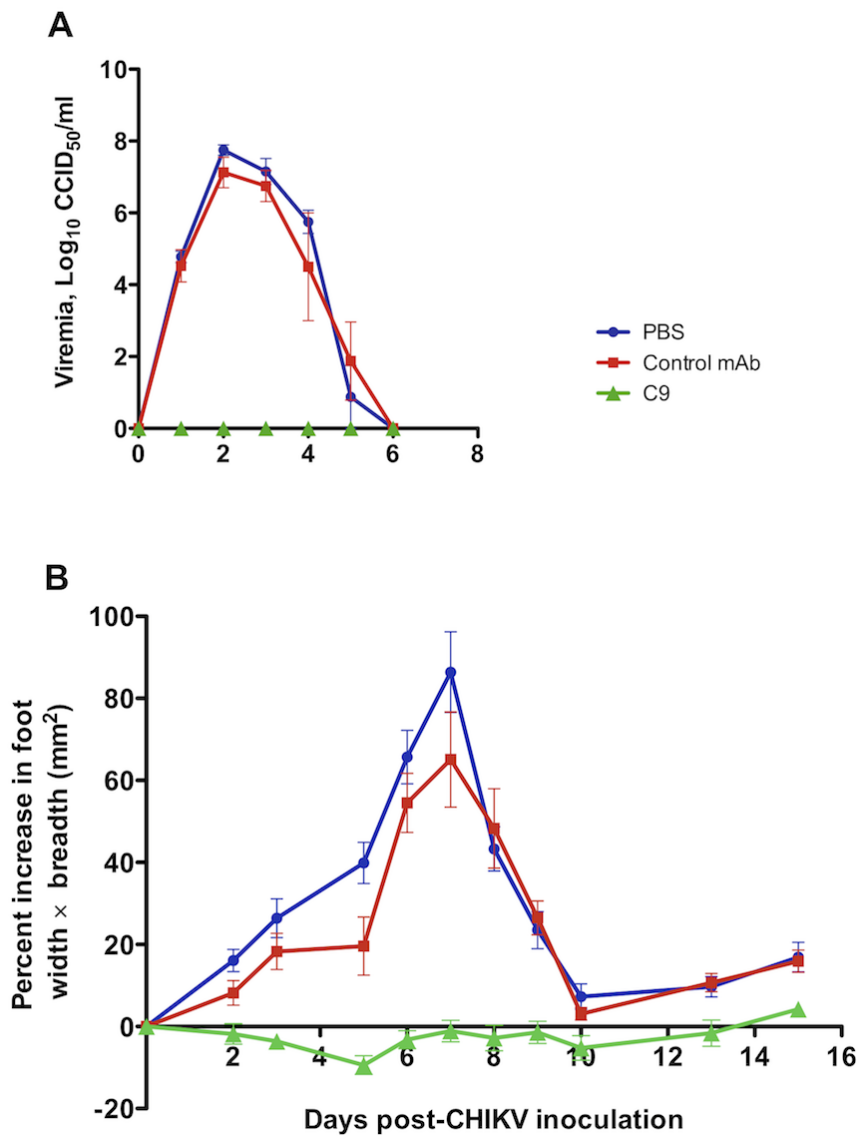
Protection against arthritis and viremia in a CHIKV mouse model. C57BL/6 mice (n = 4 mice per group) were injected with (i) PBS; (ii) purified C9 mAb; or (iii) purified control human mAb at 0.5 mg/mouse by the intraperitoneal route one day (day −1) prior to infection on day 0 with CHIKV (isolate LR2006-OPY1). (**A**) Peripheral blood viremia (CCID_50_/ml). X-axis represents days post-CHIKV inoculation; (**B**) Foot swelling over time is presented as a group average of the percentage increase in foot height×width (in the metatarsal region) for each foot compared with the same foot on day 0 (n = 8 feet).

To assess the potential protective activity of mAb C9 *in vivo*, we used an adult (6 week old) wild-type mouse model of CHIKV disease [35]. Mice received an intra-peritoneal injection of purified C9 IgG (0.5 mg/mouse or approximately 20–25 mg/kg) the day before being infected with the Reunion Island isolate of CHIKV (LR2006-OPY-1) [35]. A control monoclonal antibody that did not recognize CHIKV (produced in the same fashion as C9) and PBS were used as negative controls. Infected mice were monitored for viremia and foot swelling as described previously [35]. In both control groups, CHIKV infection of 6-week old mice resulted in a 5–6 day viremia and increased foot swelling similar to that described previously in control animals [35]. In contrast, in the same experiment, 6 week old mice injected with C9 IgG 24 hours prior to exposure to virus, showed no detectable viremia or foot swelling (Figure 5). These results demonstrate that the C9 antibody completely protected adult animals prophylactically against viremia and arthritic disease.

### C9 mAb therapeutically and prophylactically protected wild-type neonate mice

**Figure 6:**
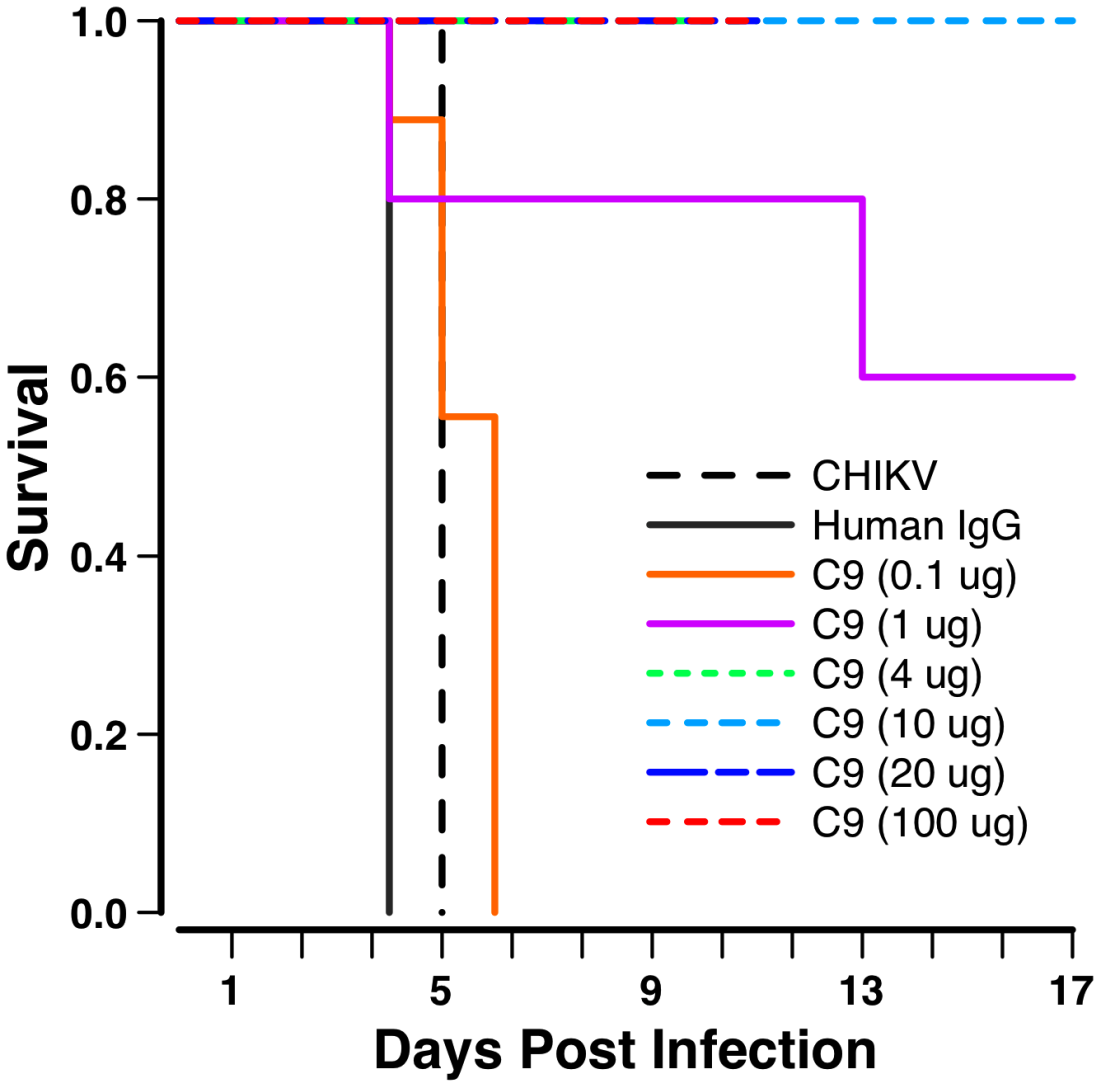
Therapeutic protection by C9 mAb of neonate mice. C57BL/6J neonatal mice were injected with 5×10^5^ PFU CHIKV (isolate S27) virus. Co-incident with infection, groups of mice (n = 5 to 8 mice per group) were inoculated with C9 mAb at the indicated concentrations or human IgG as control. The protective nature of C9 mAb at different concentrations is represented as Kaplan-Meier survival curves.

In order to evaluate the therapeutic potential of C9 mAb, we inoculated C57BL/6J neonate mice with 5×10^5^ PFU of CHIKV and monitored the survival rate. Mice infected with CHIKV survived for 5 days, while mice given control human IgG survived for 4 days (Figure 6; Table 1). In contrast 100% of the neonate mice injected with C9 at 100 µg (∼25 mg/kg) co-incident with infection survived (*P*≤0.001 compared to virus alone or human IgG). We also observed that C9 (100 µg/mouse or 25 mg/kg), when administered therapeutically at 8 hours and 18 hours after CHIKV challenge, completely protected 100% of mice (Table 1). Therefore, C9 is a potent neutralizing antibody that can therapeutically protect wild-type neonate mice from a lethal dose of virus at 8 and 18 hours post CHIKV inoculation.

**Table 1:** Therapeutic protection against CHIKV-driven lethality in neonate mice^1^.

Antibody	Antibody dose µg/mouse	Time of antibody treatment (h)	n	Percent Survival	Average Day of Death	*P*-value^2^
None	-	-	7	0	5.0	
Human IgG control	100	0	4	0	4.0	
Human IgG control	100	+18	5	20	4.25	
Human C9 mAb	100	0	12	100	-	≤0.01
Human C9 mAb	100	+8	8	100	-	≤0.01
Human C9 mAb	100	+18	7	100	-	≤0.01

1 All mice were infected with CHIKV at hour 0. Data on CHIKV infected mice without antibody treatment and mice treated with control or C9 antibody at hour 0 are also shown in Figure 6.

2 *P*-values represent all possible comparisons between C9 treated mice and untreated controls or human IgG treated animals treated with human IgG at hours 0 or +18. C9 treatment significantly affected survival in all cases.

Protection in wild-type mice from virus infection in vivo by a monoclonal neutralizing antibody is thought to require close to the IC_90_ levels of antibodies in the serum [42], [43]. However, there is very little known for chikungunya protection in mice with human anti-CHIKV mAbs. We performed *in vivo* antibody titration experiments and dosed neonates immediately before CHIKV challenge via intraperitoneal injection with C9 mAb at 100, 20, 10, 4, 1 and 0.1 µg/mouse (or approximately equaling 25, 5, 2.5, 1.0, 0.25 and 0.025 mg/kg respectively). Mice were fully protected with as little as 1 mg/kg of C9 mAb, while over 50% of mice survived after receiving 0.25 mg/kg of C9 mAb concurrent with viral challenge (Figure 6). Mice that were given 0.025 mg/kg of C9 mAb succumbed to CHIKV-driven lethality similarly to control groups. Our results demonstrate that with a potent neutralizing antibody such as C9 (IC_90_ of 0.3 µg/ml) we can protect 100% of mice with 1 mg/kg of C9 mAb and about half of all neonatal mice with only 0.25 mg/kg of C9.

## Discussion

This study describes the isolation and characterization of two human monoclonal antibodies, C9 and E8, from CHIKV infected and recovered individuals. We previously developed a CHIKV pseudovirus assay [28] that we found amenable in our current study for high-throughput screening and selection of B-cell clones expressing CHIKV neutralizing antibodies. C9 neutralizes both CHIKV pseudoviruses and replication-competent viruses with high potency. The E8 monoclonal antibody shows less dramatic neutralization of pseudovirus and does not neutralize live virus at the highest concentration tested (20 µg/ml). This suggests that although CHIKV antibody selection can be carried out using the high-throughput pseudovirus assays for greater convenience, a live virus-PRNT assay is the more reliable confirmatory assay for CHIKV neutralization.

We also report the development of a novel, comprehensive CHIKV envelope site-directed mutation library in which nearly all of the 910 residues of the full-length E1/E2 CHIKV envelope protein were individually mutated to alanine in order to identify critical amino acids that are recognized by human mAbs C9 and E8. E8 recognized 6 spatially proximal residues (Y69, F84, V113, G114, T116A and D117) in E2 domain A, however the non-neutralizing nature of the E8 antibody suggests that the residues are not easily accessible on the native CHIKV envelope on live virus, and indeed the epitope appears to be partially occluded when visualized on the native trimer crystal structure. The site-directed mutagenesis mapping studies, confirmed by neutralization escape mutant studies, revealed that E2-A162 is a critical residue required for C9 mAb recognition. Interestingly, based on the crystal structure of the CHIKV envelope, the E2-A162 residue is located in the ASR of E2 that encompasses amino acids 159–171 and 231–258 [15]. The ASR in E2, along with domain B, is a highly conserved functional region among alphaviruses and is involved in the conformational rearrangements triggered by acid pH that lead to the exposure of the fusion loop in E1 and finally results in membrane fusion [15], [40]. It is possible that neutralizing antibodies such as C9 that bind to the ASR region could fully or partially prevent the disordering and dissociation of E2 from E1 following pH triggering, thereby reducing fusion efficiency and CHIKV entry. Residues within the ASR have previously been reported to be critical for efficient particle formation and stability [44], highlighting the delicate conformational balance that this region brings to E2.

The critical E2-A162 residue is highly conserved among different CHIKV strains and is represented in the 1950's West African isolates 37997 and S27, as well as in the more recently circulating LR2006 OPY-1 strain. However, a previous study described a strain (Ag41855) isolated from Uganda during a 1982 outbreak that has a valine at the E2-162 position [41]. Mutating E2-A162 to valine did not result in a loss of C9 potency, suggesting C9 should be active against most currently circulating strains of CHIKV and other strains that arise in the future with that particular amino-acid. The fact that E2 proteins bearing the aliphatic, hydrophobic amino-acids alanine or valine did not prevent C9 neutralization, while E2 with a polar serine residue at position 162 escaped neutralization, suggests that C9 forms a critical hydrophobic interaction with that side-chain position.

Neutralizing antibodies have been shown to be critical for recovery from alphavirus infections and a number of neutralizing epitopes have been characterized, albeit only a handful for CHIKV. Of particular note, antibody R6/R13 is specific to SINV and has been previously documented to have an escape mutant at position K159N (equivalent to CHIKV residue E2-T160) in the ASR of SINV E2 glycoprotein [45]. The isolation and characterization of additional CHIKV mAbs should offer insight into the proportion of antibodies elicited against this particular epitope in CHIKV infected individuals and the timing at which they appear. For example, an elegant study recently described that a predominant proportion of the very early response to CHIKV envelope is composed of IgG3 antibodies directed against the N-terminal sequence in E2 (E2EP3) [27].

There is very little known concerning chikungunya protection with human monoclonal antibodies. In order to elucidate whether strong *in vitro* C9 neutralization would translate to protection *in vivo*, we used the C9 antibody in an adult wild-type mouse model of CHIKV disease. In contrast to control mice, mice pretreated with C9 antibody had no detectable CHIKV viremia or arthritis.

In a previous study, human IgG purified from the pooled plasma of CHIKV recovered individuals was titrated in a pathogenic neonate mouse model of chikungunya (similar to one of the models used in our current study), and was shown to fully protect only when present at a concentration of 10–25 mg per mouse [21]. Another recent study described two monoclonal antibodies (5F10 and 8B10) that were protective *in vivo* in IFN-deficient mice. These two antibodies protected 100% of the mice at ∼12.5 mg/kg (250 µg per mouse) [24]. However, the antibodies failed to protect mice at ∼1.25 mg/kg (25 µg per mouse) and were only able to delay CHIKV-driven lethality. In the *in vivo* antibody titration experiments described here we demonstrate that 100% of the mice were protected with 1 mg/kg of C9 mAb (*in vitro* IC_90_ of 0.3 µg/ml), while over 50% of mice were protected with only 0.25 mg/kg of C9. The data with C9 is consistent with what has been determined for *in vivo* protection from viral infection by neutralizing antibodies [42], while C9 at 0.25 mg/kg could also protect over half of animals from CHIKV-driven lethality.

In order to evaluate the therapeutic potential of C9 mAb we inoculated wild-type (C57BL/6J) neonate mice with a robust dose of CHIKV (5×10^5^ PFU) and monitored the survival rate. We observed that C9 mAb (100 µg/mouse), when administered therapeutically at 8 and 18 hours after CHIKV infection, completely protected 100% of mice. This report demonstrates prophylactic and therapeutic protection against viremia *in vivo* by a neutralizing antibody that targets the acid-sensitive region in CHIKV E2.

Although passive antibodies likely would not be utilized for protection from CHIKV on a regular basis, one can envisage a scenario where a potent antibody like C9 can be manufactured and used for protecting highly susceptible individuals such as pregnant women, infants and older individuals during a CHIKV epidemic, or for protecting travelers or military inadvertently exposed to the virus. While, the isolation and epitope characterization of C9 antibody and demonstration of its potent neutralization *in vitro* and *in vivo* are invaluable to future studies aimed at identification of neutralization epitopes in order to produce effective antigens for vaccines, we hypothesize that the epitope recognized by the C9 antibody is also an important region to target for antibody-based intervention in future anti-CHIKV strategies. Recent studies have demonstrated that the use of combinations of mAbs is advantageous for *in vivo* protection by limiting development of resistance [26] and different mechanisms of viral spread [24]. Given that C9 is directed against a region of CHIKV E2 not targeted by other mAbs, it will be particularly useful as a partner in such combinations.

## Supporting Information

Figure S1 **Human mAbs C9 and E8 neutralization.** Neutralization of pseudovirus bearing CHIKV LR2006-OPY-1 strain (orange lines) CHIKV S27 strain (green lines) and VSV-G control (magenta lines) envelope by (**A**) C9 or (**B**) E8 mAbs. Antibody concentration is shown in the x-axis. The results are expressed as the percentage of no antibody control and represent mean of triplicate wells, and is representative of three experiments.
(TIFF)

Figure S2 **Western blot of reduced and non-reduced CHIKV VLPs using C9 and E8 mAbs.** Antibodies (1 ug/ml) were tested for reactivity against 5 ug CHIKV VLPs that were treated with DTT/heat (+) or not (−). HRP signal was detected using luminescence by adding a 1∶1 ratio of Femto Supersignal.
(TIFF)

Text S1 **Supporting methods.**
(DOCX)
