# Supplementary figures and images for: A Neutralizing Monoclonal Antibody Targeting the Acid-Sensitive Region in Chikungunya Virus E2 Protects from Disease
Source: PLoS Negl Trop Dis. 2013 Sep 12;7(9):e2423. doi: 10.1371/journal.pntd.0002423 (PMC3772074; doi:10.1371/journal.pntd.0002423)

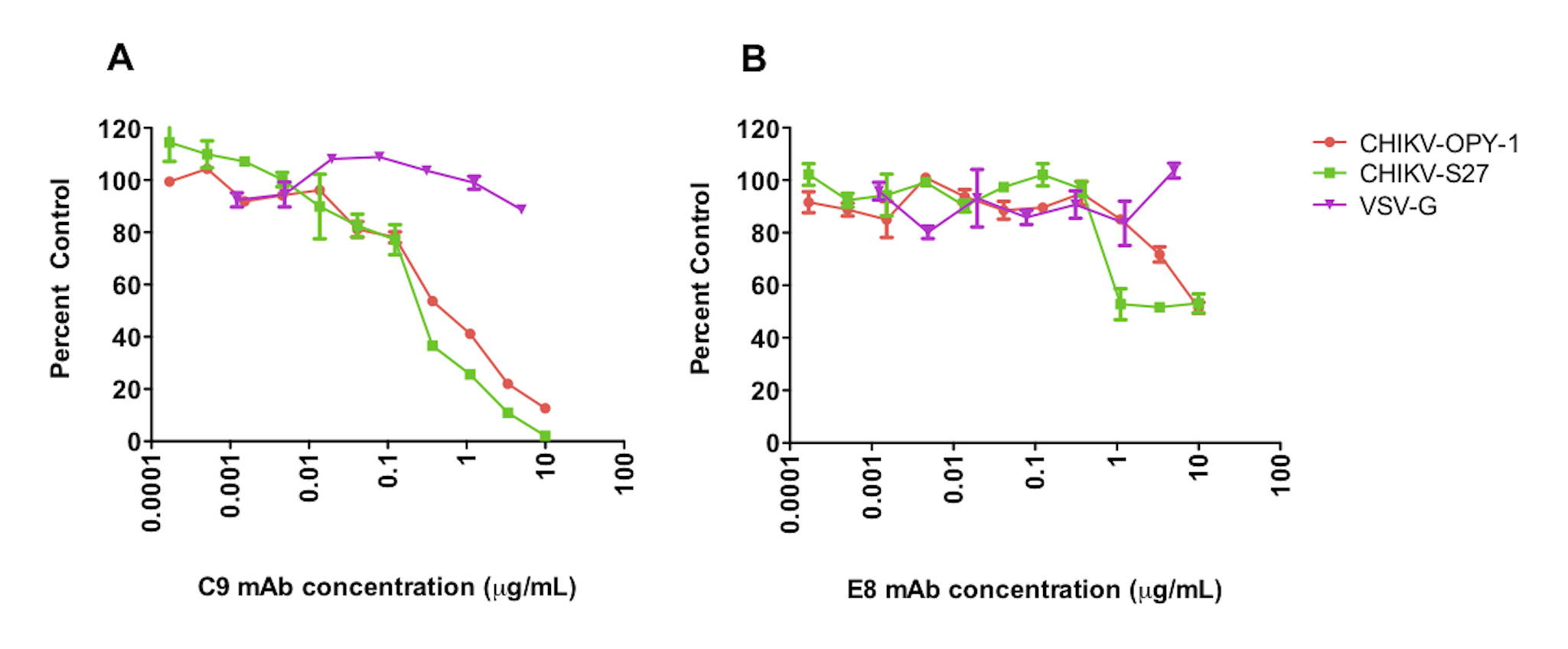

Supplement: Figure S1 — Human mAbs C9 and E8 neutralization. Neutralization of pseudovirus bearing CHIKV LR2006-OPY-1 strain (orange lines) CHIKV S27 strain (green lines) and VSV-G control (magenta lines) envelope by (A) C9 or (B) E8 mAbs. Antibody concentration is shown in the x-axis. The results are expressed as the percentage of no antibody control and represent mean of triplicate wells, and is representative of three experiments. (TIFF) [file pntd.0002423.s001.tiff]

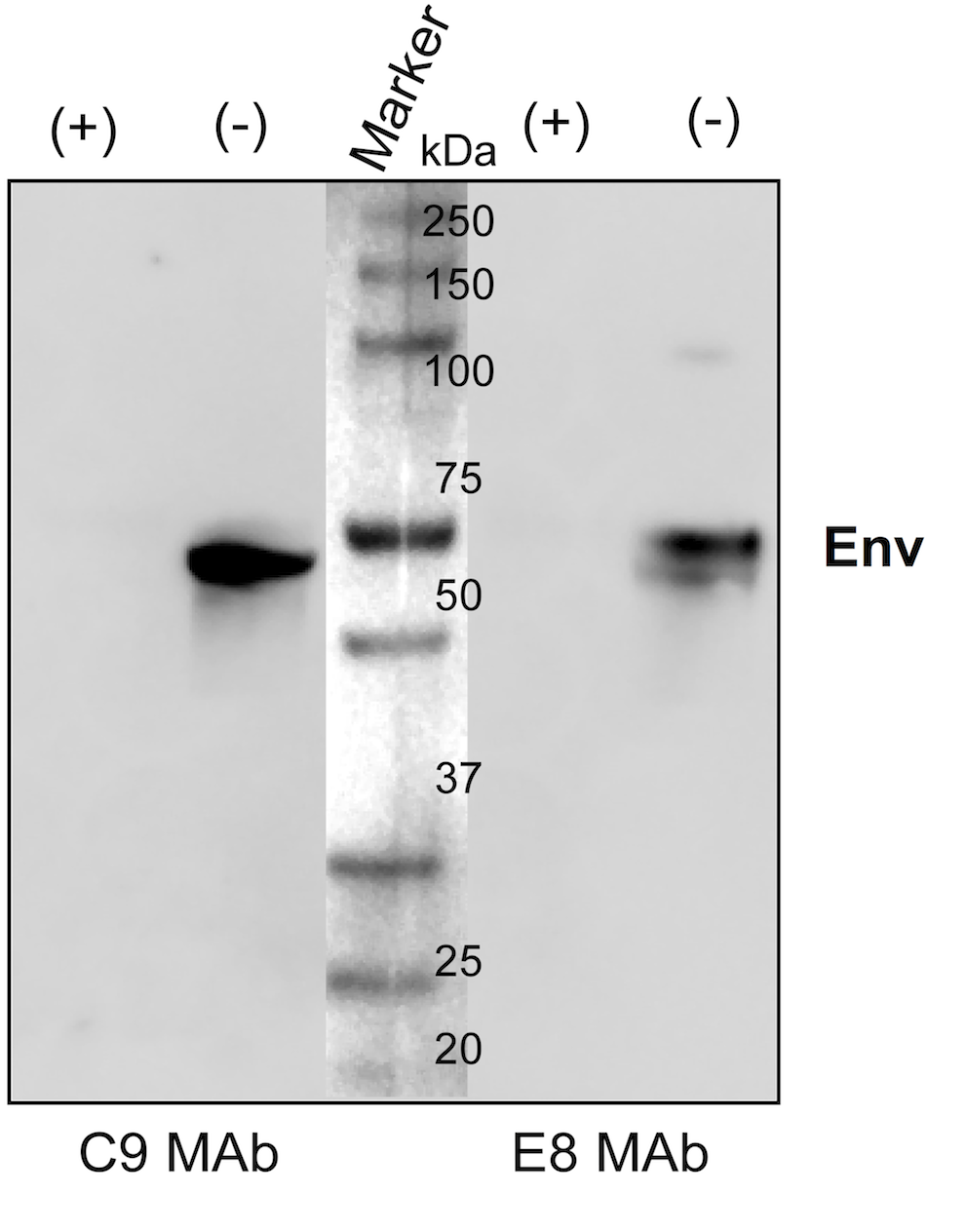

Supplement: Figure S2 — Western blot of reduced and non-reduced CHIKV VLPs using C9 and E8 mAbs. Antibodies (1 ug/ml) were tested for reactivity against 5 ug CHIKV VLPs that were treated with DTT/heat (+) or not (−). HRP signal was detected using luminescence by adding a 1∶1 ratio of Femto Supersignal. (TIFF) [file pntd.0002423.s002.tiff]
