# Supplementary material for: A Neutralizing Monoclonal Antibody Targeting the Acid-Sensitive Region in Chikungunya Virus E2 Protects from Disease
Source: PLoS Negl Trop Dis. 2013 Sep 12;7(9):e2423. doi: 10.1371/journal.pntd.0002423 (PMC3772074; doi:10.1371/journal.pntd.0002423)
Supplement: Text S1 — Supporting methods. (DOCX) [file pntd.0002423.s003.docx]

**Supporting Methods**:

**Phage display selection of CHIKV-specific FAbs using CHIKV VLPs:**

VCSM13 helper phage (Stratagene) was used to rescue the immune phage library overnight. Purified CHIKV VLPs (S27 strain) were coated directly onto a microtiter plate (Nunc) overnight at 4^o^C using 1 µg of protein per well in 0.1 M sodium bicarbonate buffer, pH 8.6. The wells were washed with phosphate-buffered saline (PBS) and the virus was pH-triggered using HEPES-buffered saline (HBS) at pH 5.8 for 10 minutes and subsequently neutralized by washing once with HBS at pH 8.0 for 1 minute. The wells were washed with PBS and blocked with 4% (w/v) non-fat dry milk in PBS (4% PBSM) for one hour. Prior to panning the CHIKV VLP coated wells, phage diluted in 4% PBSM were panned against blank wells, without antigen, to remove nonspecifically binding phage. The depleted phage were added to the CHIKV VLP coated wells and incubated for 2 hours at 37^o^C. During the first round of panning, wells were washed 3 times with PBS containing 0.01% (v/v) Tween 20 (Sigma), then once with PBS, followed by elution at 37^o^C with 10 mg/ml Trypsin (Sigma) and infection of TG1 cells. After log-phase growth, cells were infected with VCSM13 helper phage for rescue and additional selection following the same strategy as the first panning round. Three total rounds of selection were carried out with increased stringency for the second (5 washes) and third rounds (10 washes). TG1 cells were infected with the output from the third round and colonies were obtained for screening.

**Isolation and Screening of phage display FAb clones:**

Individual FAb phage clones were cultured (2xYT, 1% glucose, 50 μg/ml Carbenicillin) from single colonies isolated in the third round of panning and induced in the absence of glucose with 1 mM Isopropyl β-D-1-thiogalactopyranoside (IPTG). Culture supernatants containing FAb phage clones were screened for CHIKV-specific binding in a phage enzyme-linked immunosorbent assay (ELISA). Purified CHIKV VLPs were directly coated onto a microtiter plate overnight at 4^o^C using 0.25 µg of protein per well in 0.1 M sodium bicarbonate buffer, pH 8.6. The wells were washed with PBS and virus was treated using HBS at pH 5.8 for 10 minutes. VLPs were neutralized by washing once with HBS at pH 8.0 for 1 minute and the wells were subsequently washed with PBS and blocked with 4% PBSM for one hour. Phage clone supernatants in 2% PBSM were added to each well and the plate was incubated for one hour at 37^o^C with gentle agitation. The phage solution was discarded and the plate washed 3 times with PBS/0.01% Tween 20. To detect bound phage, a 1:5,000 dilution of horseradish peroxidase (HRP)-conjugated anti-M13 MAb (GE Healthcare) in 4% PBSM was added to the wells and incubated at 25^o^C for thirty minutes with gentle agitation. The plate was washed 3 times with PBS/0.01% Tween 20 and developed according to the manufacturer's instructions (Super Signal West Pico, Pierce). Negative controls for each clone tested included a buffer blank (no antigen, clone phages added) and a negative antigen (non-specific antigen, clone phages added). Clones that had a signal to noise greater than 4 were identified as specific for CHIKV, and DNA was isolated using the QIAprep miniprep kit (Qiagen). Phagemid DNA was sequenced with primers specific for both the light and heavy chains, and analysis of the CDR regions was performed using the programs IMGTBquest and VBase2 FAb Analysis. Families of clones were identified by grouping according to the heavy chain CDR3. Those clones which had unique CDR groupings were identified as unique, including the CAP101A.E8 (E8) clone, and the sequences were used for conversion to IgG format.
